# Supplementary material for: Patterns of Intron Gain and Loss in Fungi
Source: PLoS Biol. 2004 Nov 30;2(12):e422. doi: 10.1371/journal.pbio.0020422 (PMC532390; doi:10.1371/journal.pbio.0020422)
Supplement: Table S1 — Also available at http://genes.mit.edu/NielsenEtAl/. (4.3 MB ZIP). [file pbio.0020422.st001.zip › NielsenEtAl/html/372.html]

AN8751.1.NCU09071.1.MG02757.1.FG08635.1


```
 CLUSTAL W (1.82) Multiple Sequence Alignments - Introns Inserted


Sequence 1: NCU09071.1	701 aa
Sequence 2: MG02757.1	670 aa
Sequence 3: FG08635.1	662 aa
Sequence 4: AN8751.1	696 aa
Alignment Length: 760 aa
Number Identitical Residues: 370 aa
Alignment Score (without introns) 17232


MG02757.1 	---------MASFIGTLFPTG------KQERTERPGTPNR---NSFINPASTPQGSPSKK
NCU09071.1	---------MSSYLTNFFPSG------GKQDGTRPQTPMRPGTSSFVNPVSTPQGSPSKR
FG08635.1 	---------MASFMSSFFPGGKAANASGENSTSRPATPST-KLDNFLNPVSTPQGSPSKK
AN8751.1  	MTLNLDSKDHSNFRLPHFPPPLHTR-SGNEAFDRPSTPRD---DGFTSPISTPLGSPSKS
          	 : . .:.. :.:    **    :  : ::   ** **     ..* .* *** ***** 

MG02757.1 	TVPPGANELPNALEG-MKLVPP-------------------VLDPSIKLGRPQSVVTPLS
NCU09071.1	TLPPGANELPAAFES-MKLSTPTKLATSSRPGTSSKLGTPSALDSPIKLGRPQSVVTPLS
FG08635.1 	TNPPGAHDLPSAFESALNLNNP-------------------TIEPPLRLGRPQSVVTPLA
AN8751.1  	RMPPGALDLPSVFDNALKLQPS----------------------------------SPTK
          	  **** :** .::.:::*  .                                  :*  

MG02757.1 	PGRNNVQATDES-VFVDDSVLHKSTAAPGAHMMKQGQENTPPPLS--SARAGGGLNIAHA
NCU09071.1	PGKSNLKDVGDD-TFEDLTGNGSAVSVVHKQVDKNLPKPYPVPTSRRKERDQNQENTPPV
FG08635.1 	PGKTKTQPLDESSPNVDESVVHKSR--PGSPNKKQGQENTPPTSR---------------
AN8751.1  	NAHNNFNHPMFTLPNQSGETFNESVIRPASPSRRPKNENTLPQSAP--------------
          	 .:.: :         .     .:         :   :                      

MG02757.1 	HT-------DQQAAPSHN--HAAVSRHEHY-LRDRPTTPATRRFNTNRGLTPEELEILKK
NCU09071.1	VTRDPRDPRDPATEPTYQRSHAALSRQELY-QARERASPTVKRFNTSRGLTPEERELLNK
FG08635.1 	---------LATLESPHQHSHAAVTRQQLYEQRDRPTTPATKRFNTHRGLTAEEREILQK
AN8751.1  	----------LTKDLGRNPAAAAISRQEQY--HTRDLESVQRRQVPMRGLTPEELEKLQN
          	                 : : **::*:: *    .   .. :*  . ****.** * *::

MG02757.1 	PSIKRLVNVTQLY1FLDYYFDLLTYTGARQNRLAAFKSEYPPPPETDEETYAAMWHKYTG
NCU09071.1	PNVKRLVNVTQLY1FLDYYFDLLTYVGQRQNRLNAFKAEFPPPPETDEETYKQMWHKYAG
FG08635.1 	PNVKRLVNVTQLY1FLDYYFDLLTYVGARQNRLQAFKNEYPPPPETDEQTHNQMWAKYAG
AN8751.1  	PRVKRLVNVTQLY1FLDYYFDLLSYVHNRQTRYSQFCAANPEPPATPIEEYEPALSKYLG
          	* :********** *********:*.  **.*   *    * ** *  : :     ** *

MG02757.1 	RERANLRKRRVRLRQGDFQILTQVGQGGYGQVFLAQKKDTREVCALKVMSKKLLFKLDEV
NCU09071.1	RERANLRKRRVRLRQGDFQILTQVGQGGYGQVFLAQKKDTKEVCALKVMSKKLLFKLDEV
FG08635.1 	RERANLRKRRVRLRHGDFQILTQVGQGGYGQVFLAQKKDTREVCALKVMSKKLLFKLDEV
AN8751.1  	RERAHLRKRRTRLRQGDFQILTQVGQGGYGQVYLASKKDTKEVCALKVMSKKLLFKLDEI
          	****:*****.***:*****************:**.****:******************:

MG02757.1 	RHVLTERDILTTAKSEWLVRLLYSFQDDKSIYLAM0EYVPGGDFRTLLNNTGVLSNRHAR
NCU09071.1	RHVLTERDILTTAKSDWLVRLLYSFQDDKNIYLAM~EYVPGGDFRTLLNNTGVLSNRHAR
FG08635.1 	RHVLTERDILTTAQSEWLVRLLYSFQDERSIYLAM~EYVPGGDFRTLLNNTGVLSNRHAR
AN8751.1  	RHILTERDILTAAKSEWLVKLLYAFQDETQIYFAM~EFVPGGDFRTLLNNTGVLHNRHAR
          	**:********:*:*:***:***:***: .**:** *:**************** *****

MG02757.1 	FYIAEMFCSVDALHQLGYIHRDLKPENFLVDSTGHVKLTDFGLAAGVLAPGKIESMRLRL
NCU09071.1	FYIAEMFCAVDALHQLGYIHRDLKPENFLVDSTGHVKLTDFGLAAGFLAPAKIESMRIRL
FG08635.1 	FYIAEMFCSVDALHQLGYIHRDLKPENFLVDSTGHIKLTDFGLAAGVLAPSKIESMRVKL
AN8751.1  	FYIAEMFCCIDALHALGYIHRDLKPENFLIDSTGHVKLTDFGLAAGMLNPRKIESMRVKL
          	********.:**** **************:*****:**********.* * ******::*

MG02757.1 	EKASETSVPFGKPMDQRTIAERREGYRSMRDKDVNYAKSIVGSPDYMAPEVLRGEEYDFT
NCU09071.1	EKASETSVPFGKPMDQRTVAERREGYRTMRDRDTNYAKSIVGSPDYMAPEVLRGEEYDYT
FG08635.1 	EKASESSVPFGKPMDQRTVAERRESYRTMRENDVNYAKSIVGSPDYMAPEVLRGEEYDFT
AN8751.1  	EEVGNTPVPFGRPMEQRTMAERRQGYRSLRERQVDYAKSIVGSPDYMAPEVLKGEEYDFT
          	*:..::.****:**:***:****:.**::*:.:.:*****************:*****:*

MG02757.1 	VDYWSLGCMLFEALTGFPPFAGATAEETWRNLKHWKEVLKRPVWEDPNYFLSNRTWGFIT
NCU09071.1	VDYWSLGCMLFEALTGFPPFAGSTADETWRNLKHWKEVLKRPVWEDPNYFLSNRTWSFIT
FG08635.1 	VDYWSLGCMLFEALTGFPPFAGATPDETWRNLKHWKEVLKRPVWEDPNYFLSNRTWNFIC
AN8751.1  	VDYWSLGCMLFEALAGYPPFAGSTVDETWQNLKNWQKVLRRPVYEDPNYFLSKRTWNFIT
          	**************:*:*****:* :***:***:*::**:***:********:***.** 

MG02757.1 	T~CINSRSRRFANIRDIYAHQYFAEVDWETLRETKAPFVPELDSETDAGYFDDFSNEADM
NCU09071.1	T2CINSRSRRFNNIKDIYAHHYFAEVDWAILRETKAPFVPELDSETDAGYFDDFSNEADM
FG08635.1 	T2CINSRSRRFSNITDIFAHQYFAEVEWDVLRQTRAPFVPELDSETDAGYFDDFTNEADM
AN8751.1  	T~LVASREHRFKNISEIHAHEYFSEVDFSRLREQRPPFVPQLDSETDAGYFDDFENEADM
          	*  : **.:** ** :*.**.**:**::  **: :.****:************* *****

MG02757.1 	AKYKEVHEKQQALETMAERDDEMGKSLFVGFTFRHR-----------KPQGEDSSSPRKP
NCU09071.1	AKYKEVHEKQQALEGLAERGEEMNKSLFVGFTFRHR-----------KPATDDGASPRKP
FG08635.1 	AKYKEVHDKQQALETMAEREDQMSKSLFVGFTFRHR-----------KPATEDGASPRKR
AN8751.1  	AKYKEVHDKQRALEEMAERDEKMAKGLFVGFTFRFVPCFLLVNDESNQLQTSQAEHRRNW
          	*******:**:*** :*** ::* *.********. ..    ...:.:   .:.   *: 

MG02757.1 	LTADTFQAETFGTML----------------------------
NCU09071.1	IPFE--ESNNFGTML----------------------------
FG08635.1 	IPTD----ESFGTMF----------------------------
AN8751.1  	ASLSPQADTNRGLWYYLLGLMTLLSPLSLLWSGQAGLEWPLSD
          	 . ..    . *      .  :  :. :   :..:. . . :.
```
